# Diaqua­dichloridobis[quinazolin-4(1*H*)-one-κ*N* ^3^]nickel(II)

**DOI:** 10.1107/S1600536812019381

**Published:** 2012-05-05

**Authors:** Shirin Shomurotova, Kambarali K. Turgunov, Nasir Mukhamedov, Bakhodir Tashkhodjaev

**Affiliations:** a Tashkent State Pedagogical University Named After Nizami, Yusuf Khos Khojib Str 103, Tashkent 100100, Uzbekistan; b S. Yunusov Institute of the Chemistry of Plant Substances, Academy of Sciences of Uzbekistan, Mirzo Ulugbek Str 77, Tashkent 100170, Uzbekistan

## Abstract

In the title complex, [NiCl_2_(C_8_H_6_N_2_O)_2_(H_2_O)_2_], the Ni^II^ ion is located on an inversion center and is six-coordinated by two N atoms of 1*H*-quinazolin-4-one ligands, two chloride ions and two water mol­ecules. The water mol­ecules are involved in intra- and inter­molecular O—H⋯O and O—H⋯Cl hydrogen bonding. Inter­molecular N—H⋯O and N—H⋯Cl hydrogen bonds are formed between ligands. In addition, weak π–π inter­actions are observed between the benzene rings of the ligands [centroid–centroid distance = 3.580 (3) Å]. The inter­molecular hydrogen bonds and π–π inter­actions lead to the formation of a three-dimensional supra­molecular network.

## Related literature

For a Cd(II) coordination polymer with quinazolin-4(3*H*)-one, see: Turgunov & Englert (2010 ▶) and for a Cu(II) coordination compound with quinazolin-4(1*H*)-one, see: Turgunov *et al.* (2010 ▶).
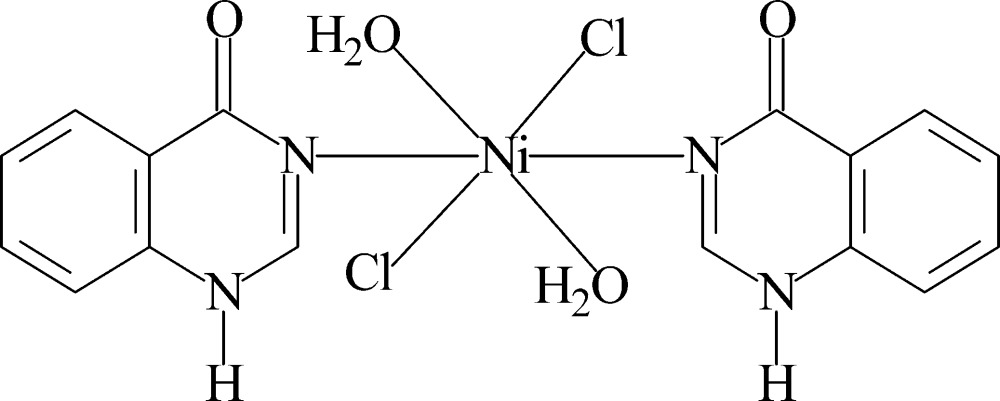


## Experimental

### 

#### Crystal data

[NiCl_2_(C_8_H_6_N_2_O)_2_(H_2_O)_2_]
*M* *_r_* = 457.94
Monoclinic, 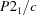

*a* = 6.7800 (5) Å
*b* = 18.741 (2) Å
*c* = 6.6106 (5) Å
β = 93.782 (8)°
*V* = 838.14 (13) Å^3^
*Z* = 2
Cu *K*α radiation
μ = 4.92 mm^−1^
*T* = 295 K
0.16 × 0.16 × 0.04 mm

#### Data collection

Oxford Diffraction Xcalibur Ruby diffractometer
Absorption correction: multi-scan (*CrysAlis PRO*; Oxford Diffraction, 2009 ▶) *T*_min_ = 0.621, *T*_max_ = 1.000
3040 measured reflections
1686 independent reflections
1046 reflections with *I* > 2σ(*I*)
*R*_int_ = 0.047

#### Refinement

*R*[*F* ^2^ > 2σ(*F* ^2^)] = 0.052
*wR*(*F* ^2^) = 0.139
*S* = 0.94
1686 reflections
132 parameters
2 restraints
H atoms treated by a mixture of independent and constrained refinement
Δρ_max_ = 0.75 e Å^−3^
Δρ_min_ = −0.51 e Å^−3^

### 

Data collection: *CrysAlis PRO* (Oxford Diffraction, 2009 ▶); cell refinement: *CrysAlis PRO*; data reduction: *CrysAlis PRO*; program(s) used to solve structure: *SHELXS97* (Sheldrick, 2008 ▶); program(s) used to refine structure: *SHELXL97* (Sheldrick, 2008 ▶); molecular graphics: *XP* in *SHELXTL* (Sheldrick, 2008 ▶); software used to prepare material for publication: *publCIF* (Westrip, 2010 ▶).

## Supplementary Material

Crystal structure: contains datablock(s) I, global. DOI: 10.1107/S1600536812019381/hg5221sup1.cif

Structure factors: contains datablock(s) I. DOI: 10.1107/S1600536812019381/hg5221Isup2.hkl

Additional supplementary materials:  crystallographic information; 3D view; checkCIF report

## Figures and Tables

**Table 1:** Hydrogen-bond geometry (Å, °)

*D*—H⋯*A*	*D*—H	H⋯*A*	*D*⋯*A*	*D*—H⋯*A*
O1*W*—H1*W*⋯Cl1^i^	0.85 (4)	2.56 (3)	3.371 (4)	160 (6)
O1*W*—H2*W*⋯O1^ii^	0.85 (5)	1.87 (6)	2.641 (5)	150 (11)
N1—H1*A*⋯O1^iii^	0.86	2.44	3.116 (5)	136
N1—H1*A*⋯Cl1^iv^	0.86	2.59	3.256 (4)	135
C2—H2*A*⋯O1*W*	0.93	2.42	2.958 (6)	117

Symmetry codes: (i) 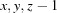; (ii) 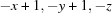; (iii) 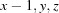; (iv) 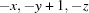.

**Fig. 1.**
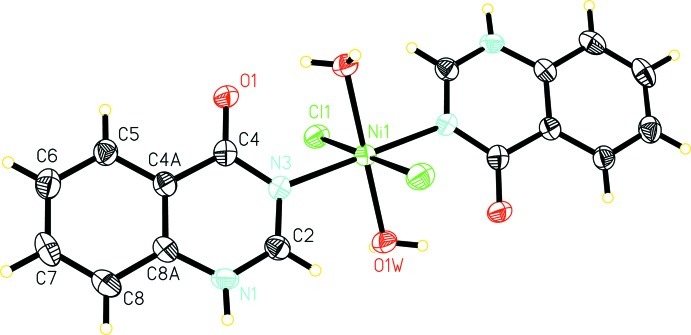
The molecular structure of the title complex with the atom-numbering scheme. Displacement ellipsoids are drawn at the 50% probability level.

**Fig. 2.**
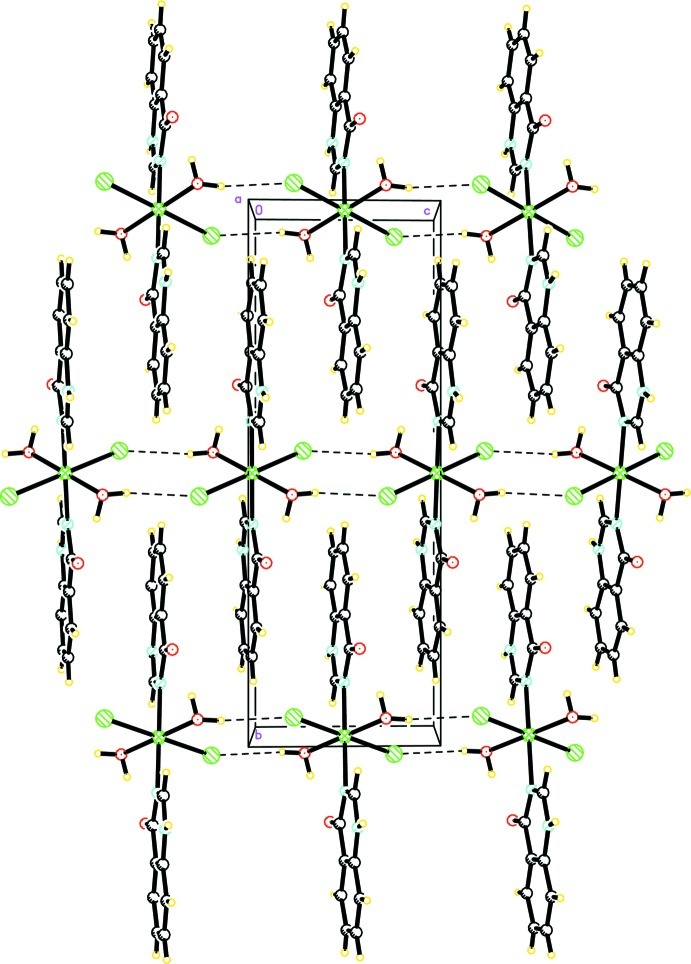
Crystal packing of the title compound viewed along the a direction showing the formation a hydrogen-bonded chain along [001].

**Fig. 3.**
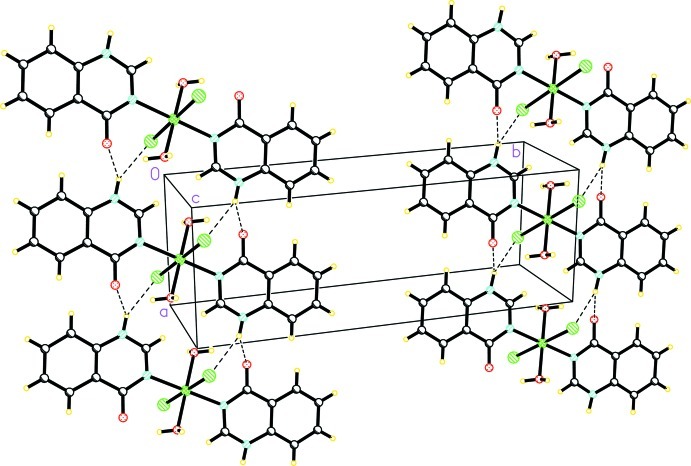
Part of the crystal structure of the title compound showing the formation a hydrogen-bonded chain along [100].

## supplementary crystallographic information

### Comment

In the title compound Ni^II^ ion is located on the inversion center and has an octahedral coordination enviroment: two ligands coordinated *via* N atoms, two chloride ligands and two aqua ligands (Figure 1). The distances between Ni and coordination atoms are the following: d(Ni–N3)= 2.112 (4) Å, d(Ni–Cl)=2.445 (1) Å, d(Ni–Ow)=2.084 (3) Å. In the isostructural Cu^II^ complex Cu–Ow distance was longer (2.512 Å) because of the Jahn-Teller elongation effect (Turgunov *et al.*, 2010).

The flat quinazolinone ligand is a little tilted in respect to metal–nitrogen vector and the dihedral angle between the least squares plane through the ligand and the metal–halide–water plane amounts to 84.33 (9)°.

Aqua ligands are involved in intramolecular and intermolecular hydrogen bonding. Intramolecular H-bonding is occurring with carbonyl group of the ligand. An intermolecular H-bonding of aqua and chloride ligands gives raise to chains along [001] (Figure 2). In addition, between ligand and water molecules are formed weak C—H···O hydrogen bonds. Intermolecular N—H···O and N—H···Cl hydrogen bonds formed between the organic and chloride ligands link molecular complexes into hydrogen-bonded chains along [100] (Figure 3; Table 1). Weak π-π ring interactions connect the molecular complexes along [010] and [001] directions. [*Cg*1···*Cg*1^v^]=3.580 Å, where *Cg*1=C4AC5C6C7C8C8A; ^v^=*x, 3/2 - y,1/2 + z*].

### Experimental

A solution of 23.77 mg (0.1 mmol) of nickel(II) chloride hexahydrate in 1 ml of water was added to a solution of 29.23 mg (0.2 mmol) of 3*H*-quinazolin-4-one in 3 ml of ethanol. The solution allowed to stand at 50° C temperature for one week, after which colourless crystals were obtained.

### Refinement

Ligand H atoms were positioned geometrically and treated as riding on their C and N atoms, with C—H distances of 0.93 Å (aromatic), N—H distance of 0.86 Å and were refined with *U*_iso_(H)=1.2Ueq(C),*U*_iso_(H)=1.2Ueq(N). Coordinated water H atoms were found by difference Fourier synthesis and refined isotropically with distance restrains of 0.85 Å [O1w—H1w =0.85 (4) Å, O1w—H2w = 0.85 (5) Å].

### Crystal data

**Table:**

[NiCl_2_(C_8_H_6_N_2_O)_2_(H_2_O)_2_]	*F*(000) = 468
*M**_r_* = 457.94	*D*_x_ = 1.815 Mg m^−^^3^
Monoclinic, *P*2_1_/*c*	Cu *K*α radiation, λ = 1.54184 Å
Hall symbol: -P 2ybc	Cell parameters from 792 reflections
*a* = 6.7800 (5) Å	θ = 4.7–75.6°
*b* = 18.741 (2) Å	µ = 4.92 mm^−^^1^
*c* = 6.6106 (5) Å	*T* = 295 K
β = 93.782 (8)°	Rhombic plates, colourless
*V* = 838.14 (13) Å^3^	0.16 × 0.16 × 0.04 mm
*Z* = 2	

### Data collection

**Table:**

Oxford Diffraction Xcalibur Ruby diffractometer	1686 independent reflections
Radiation source: Enhance (Cu) X-ray Source	1046 reflections with *I* > 2σ(*I*)
Graphite monochromator	*R*_int_ = 0.047
Detector resolution: 10.2576 pixels mm^-1^	θ_max_ = 75.9°, θ_min_ = 4.7°
ω scans	*h* = −8→7
Absorption correction: multi-scan (*CrysAlis PRO*; Oxford Diffraction, 2009)	*k* = −23→13
*T*_min_ = 0.621, *T*_max_ = 1.000	*l* = −8→8
3040 measured reflections	

### Refinement

**Table:**

Refinement on *F*^2^	Primary atom site location: structure-invariant direct methods
Least-squares matrix: full	Secondary atom site location: difference Fourier map
*R*[*F*^2^ > 2σ(*F*^2^)] = 0.052	Hydrogen site location: inferred from neighbouring sites
*wR*(*F*^2^) = 0.139	H atoms treated by a mixture of independent and constrained refinement
*S* = 0.94	*w* = 1/[σ^2^(*F*_o_^2^) + (0.0729*P*)^2^] where *P* = (*F*_o_^2^ + 2*F*_c_^2^)/3
1686 reflections	(Δ/σ)_max_ < 0.001
132 parameters	Δρ_max_ = 0.75 e Å^−^^3^
2 restraints	Δρ_min_ = −0.51 e Å^−^^3^

### Special details

**Table:**

Geometry. All e.s.d.'s (except the e.s.d. in the dihedral angle between two l.s. planes) are estimated using the full covariance matrix. The cell e.s.d.'s are taken into account individually in the estimation of e.s.d.'s in distances, angles and torsion angles; correlations between e.s.d.'s in cell parameters are only used when they are defined by crystal symmetry. An approximate (isotropic) treatment of cell e.s.d.'s is used for estimating e.s.d.'s involving l.s. planes.
Refinement. Refinement of *F*^2^ against ALL reflections. The weighted *R*-factor *wR* and goodness of fit *S* are based on *F*^2^, conventional *R*-factors *R* are based on *F*, with *F* set to zero for negative *F*^2^. The threshold expression of *F*^2^ > σ(*F*^2^) is used only for calculating *R*-factors(gt) *etc*. and is not relevant to the choice of reflections for refinement. *R*-factors based on *F*^2^ are statistically about twice as large as those based on *F*, and *R*- factors based on ALL data will be even larger.

### Fractional atomic coordinates and isotropic or equivalent isotropic displacement parameters (Å^2^)

**Table:**

	*x*	*y*	*z*	*U*_iso_*/*U*_eq_	
Ni1	0.5000	0.5000	0.0000	0.0271 (3)	
Cl1	0.30215 (17)	0.45677 (7)	0.27085 (17)	0.0340 (3)	
O1	0.6109 (5)	0.66887 (19)	0.0800 (5)	0.0340 (8)	
N1	0.0343 (5)	0.6502 (2)	−0.0692 (6)	0.0301 (9)	
H1A	−0.0903	0.6434	−0.0945	0.036*	
C2	0.1533 (6)	0.5947 (3)	−0.0494 (6)	0.0285 (10)	
H2A	0.0971	0.5498	−0.0684	0.034*	
N3	0.3448 (5)	0.5977 (2)	−0.0048 (5)	0.0272 (9)	
C4	0.4314 (7)	0.6634 (3)	0.0282 (7)	0.0272 (10)	
C4A	0.3084 (7)	0.7271 (3)	−0.0011 (7)	0.0268 (10)	
C5	0.3861 (7)	0.7962 (3)	0.0203 (7)	0.0299 (10)	
H5A	0.5200	0.8025	0.0561	0.036*	
C6	0.2672 (8)	0.8542 (3)	−0.0110 (7)	0.0348 (12)	
H6A	0.3205	0.8999	0.0004	0.042*	
C7	0.0666 (8)	0.8454 (3)	−0.0599 (7)	0.0368 (12)	
H7A	−0.0131	0.8855	−0.0793	0.044*	
C8	−0.0178 (8)	0.7785 (3)	−0.0805 (8)	0.0357 (12)	
H8A	−0.1525	0.7732	−0.1134	0.043*	
C8A	0.1054 (7)	0.7188 (3)	−0.0504 (7)	0.0283 (10)	
O1W	0.2955 (5)	0.4596 (2)	−0.2198 (5)	0.0303 (7)	
H1W	0.328 (10)	0.463 (4)	−0.342 (4)	0.08 (2)*	
H2W	0.282 (18)	0.4160 (17)	−0.189 (17)	0.20 (6)*	

### Atomic displacement parameters (Å^2^)

**Table:**

	*U*^11^	*U*^22^	*U*^33^	*U*^12^	*U*^13^	*U*^23^
Ni1	0.0258 (5)	0.0247 (6)	0.0301 (6)	−0.0004 (5)	−0.0038 (4)	−0.0003 (5)
Cl1	0.0330 (6)	0.0362 (7)	0.0327 (6)	−0.0058 (5)	0.0008 (4)	0.0007 (5)
O1	0.0234 (15)	0.029 (2)	0.049 (2)	−0.0023 (15)	−0.0044 (14)	−0.0022 (17)
N1	0.0206 (17)	0.035 (2)	0.034 (2)	−0.0025 (17)	−0.0034 (15)	0.0038 (19)
C2	0.029 (2)	0.031 (3)	0.026 (2)	0.001 (2)	0.0016 (18)	0.002 (2)
N3	0.0273 (19)	0.023 (2)	0.031 (2)	−0.0022 (17)	0.0002 (15)	0.0001 (17)
C4	0.031 (2)	0.025 (3)	0.025 (2)	0.001 (2)	0.0043 (18)	0.000 (2)
C4A	0.028 (2)	0.026 (3)	0.026 (2)	0.002 (2)	0.0020 (18)	0.002 (2)
C5	0.036 (2)	0.026 (3)	0.027 (2)	−0.001 (2)	−0.0003 (19)	0.002 (2)
C6	0.048 (3)	0.028 (3)	0.029 (2)	0.001 (2)	0.000 (2)	0.000 (2)
C7	0.050 (3)	0.032 (3)	0.029 (2)	0.017 (3)	0.002 (2)	0.003 (2)
C8	0.032 (2)	0.040 (3)	0.034 (3)	0.011 (2)	−0.002 (2)	0.001 (2)
C8A	0.029 (2)	0.029 (3)	0.027 (2)	0.005 (2)	0.0014 (18)	0.002 (2)
O1W	0.0299 (16)	0.031 (2)	0.0292 (17)	−0.0041 (16)	−0.0033 (13)	−0.0022 (17)

### Geometric parameters (Å, º)

**Table:**

Ni1—O1W	2.084 (3)	C4—C4A	1.462 (7)
Ni1—O1W^i^	2.084 (3)	C4A—C8A	1.402 (6)
Ni1—N3^i^	2.112 (4)	C4A—C5	1.403 (7)
Ni1—N3	2.112 (4)	C5—C6	1.362 (7)
Ni1—Cl1	2.4451 (12)	C5—H5A	0.9300
Ni1—Cl1^i^	2.4451 (12)	C6—C7	1.387 (7)
O1—C4	1.246 (5)	C6—H6A	0.9300
N1—C2	1.317 (6)	C7—C8	1.381 (8)
N1—C8A	1.375 (6)	C7—H7A	0.9300
N1—H1A	0.8600	C8—C8A	1.402 (7)
C2—N3	1.314 (5)	C8—H8A	0.9300
C2—H2A	0.9300	O1W—H1W	0.85 (4)
N3—C4	1.374 (6)	O1W—H2W	0.85 (5)
			
O1W—Ni1—O1W^i^	180.0 (2)	O1—C4—N3	121.1 (5)
O1W—Ni1—N3^i^	90.21 (15)	O1—C4—C4A	120.5 (5)
O1W^i^—Ni1—N3^i^	89.79 (15)	N3—C4—C4A	118.4 (4)
O1W—Ni1—N3	89.79 (15)	C8A—C4A—C5	118.8 (5)
O1W^i^—Ni1—N3	90.21 (15)	C8A—C4A—C4	119.0 (5)
N3^i^—Ni1—N3	180.0 (2)	C5—C4A—C4	122.2 (4)
O1W—Ni1—Cl1	91.05 (11)	C6—C5—C4A	120.5 (5)
O1W^i^—Ni1—Cl1	88.95 (11)	C6—C5—H5A	119.8
N3^i^—Ni1—Cl1	89.91 (11)	C4A—C5—H5A	119.8
N3—Ni1—Cl1	90.09 (11)	C5—C6—C7	120.1 (5)
O1W—Ni1—Cl1^i^	88.95 (11)	C5—C6—H6A	119.9
O1W^i^—Ni1—Cl1^i^	91.05 (11)	C7—C6—H6A	119.9
N3^i^—Ni1—Cl1^i^	90.09 (11)	C8—C7—C6	121.6 (5)
N3—Ni1—Cl1^i^	89.91 (11)	C8—C7—H7A	119.2
Cl1—Ni1—Cl1^i^	180.00 (6)	C6—C7—H7A	119.2
C2—N1—C8A	121.4 (4)	C7—C8—C8A	118.1 (5)
C2—N1—H1A	119.3	C7—C8—H8A	120.9
C8A—N1—H1A	119.3	C8A—C8—H8A	120.9
N3—C2—N1	125.3 (5)	N1—C8A—C8	122.1 (5)
N3—C2—H2A	117.3	N1—C8A—C4A	117.2 (4)
N1—C2—H2A	117.3	C8—C8A—C4A	120.8 (5)
C2—N3—C4	118.7 (4)	Ni1—O1W—H1W	115 (5)
C2—N3—Ni1	116.8 (3)	Ni1—O1W—H2W	105 (8)
C4—N3—Ni1	124.5 (3)	H1W—O1W—H2W	110 (8)

Symmetry code: (i) −*x*+1, −*y*+1, −*z*.

### Hydrogen-bond geometry (Å, º)

**Table:**

*D*—H···*A*	*D*—H	H···*A*	*D*···*A*	*D*—H···*A*
O1*W*—H1*W*···Cl1^ii^	0.85 (4)	2.56 (3)	3.371 (4)	160 (6)
O1*W*—H2*W*···O1^i^	0.85 (5)	1.87 (6)	2.641 (5)	150 (11)
N1—H1*A*···O1^iii^	0.86	2.44	3.116 (5)	136
N1—H1*A*···Cl1^iv^	0.86	2.59	3.256 (4)	135
C2—H2*A*···O1*W*	0.93	2.42	2.958 (6)	117

Symmetry codes: (i) −*x*+1, −*y*+1, −*z*; (ii) *x*, *y*, *z*−1; (iii) *x*−1, *y*, *z*; (iv) −*x*, −*y*+1, −*z*.
